# Cholesterol, high-density lipoprotein, and glucose index is associated with renal survival in patients with IgA nephropathy

**DOI:** 10.3389/fendo.2025.1643360

**Published:** 2025-10-01

**Authors:** Hong Liu, Guijing Tang, Yuan Yuan, Peng Gu, Anni Wang, Shuman Zhao, Xue Jiang

**Affiliations:** Department of Nephrology, Hangzhou Traditional Chinese Medicine (TCM) Hospital Affiliated to Zhejiang Chinese Medical University, Hangzhou, Zhejiang, China

**Keywords:** the cholesterol, high-density lipoprotein, and glucose (CHG) index, IgA nephropathy, insulin resistance (IR), renal survival, prognosis

## Abstract

**Background:**

The cholesterol, high-density lipoprotein, and glucose (CHG) index has emerged as a novel biomarker for insulin resistance (IR) and metabolic syndrome (MS). However, its prognostic role in IgA nephropathy (IgAN) remains underexplored. This study investigated the association between the CHG index and IgAN and aimed to clarify the importance of the CHG index in evaluating the prognosis of IgAN.

**Methods:**

This single-centre retrospective study included 1791 patients with biopsy-confirmed IgAN who had more than 12 months of follow-up from October 2014 to September 2023. The optimal CHG index cut-off for renal endpoints was determined using receiver operating characteristic (ROC) analysis. Associations were assessed via correlation analysis, Cox regression, and Kaplan–Meier survival analysis.

**Results:**

Patients were stratified into three baseline CHG index tertiles. High baseline CHG index was correlated with adverse clinicopathological features and reduced renal survival (Kaplan–Meier, log-rank=26.51; P<0.001). Correlation analyses revealed that the CHG index was positively associated with tubular atrophy/interstitial fibrosis, as well as intimal thickening and hyaline degeneration of renal arteries (P<0.001). The optimal CHG index cut-off for renal survival was 5.29 (sensitivity=63.1%, specificity=63.0%, AUC = 0.646), and patients were divided into a low group (CHG index ≤ 5.29; n=1,096) and a high group (CHG index >5.29; n=695). Subgroup analysis revealed that elevated CHG indices had a more pronounced prognostic value for renal outcomes in subgroups of patients with a BMI ≤ 24.9, a 24-hour urine protein level ≤ 3.5 g, an estimated glomerular filtration rate (eGFR)≤45 mL/min/1.73m², and an Oxford renal pathology classification (MEST-C)T0. Multivariate Cox regression analysis demonstrated that elevated CHG indices were significantly associated with worse renal outcomes in patients with IgAN (adjusted HR: 1.842; 95% CI: 1.044–3.249; P = 0.035).

**Conclusion:**

Elevated CHG index is an independent risk factor for predicting renal prognosis in IgAN. Compared with patients with low CHG indices, those with CHG indices > 5.29 have an 84.2% greater risk of poor renal outcomes.

## Introduction

IgA nephropathy (IgAN), the most common type of primary glomerulonephritis (GN) worldwide, is histopathologically characterized by dominant mesangial IgA deposition on renal biopsy (1). It represents a leading cause of end-stage renal disease (ESRD), with an estimated 25–50% of patients progressing to ESRD within 20 to 30 years after diagnosis (2, 3). Currently, there is a lack of definitive and reliable prognostic markers for IgAN. Although the International IgAN Prediction Tool (IIgAN-PT model), endorsed by the 2021 clinical guidelines (4), provides risk estimates for adverse renal outcomes within 5–7 years after biopsy, the inherent subjectivity and considerable interobserver variability of the Oxford Classification (MEST-C) histological scoring system have limited its widespread clinical adoption. Thus, robust screening strategies to facilitate early identification of high-risk patients with IgAN and reduce the incidence of ESRD are urgently needed.

Metabolic syndrome (MS) is a cluster of metabolic abnormalities—including obesity, hypertension, hyperglycaemia, and dyslipidaemia—driven primarily by insulin resistance (IR) (5). Accumulating evidence indicates that both MS and IR are significantly associated with renal function decline and poor outcome in patients with IgAN (6, 7). The CHG index, initially proposed by Mansoori et al. (8) for the diagnosis of type 2 diabetes, has recently emerged as a novel biomarker of metabolic dysfunction and IR (9). Nonetheless, its relationship with disease progression in IgAN remains unexplored. Therefore, this study aimed to evaluate the potential association between the CHG index and renal survival in patients with IgAN.

## Material and methods

### Study population

**Figure 1:**
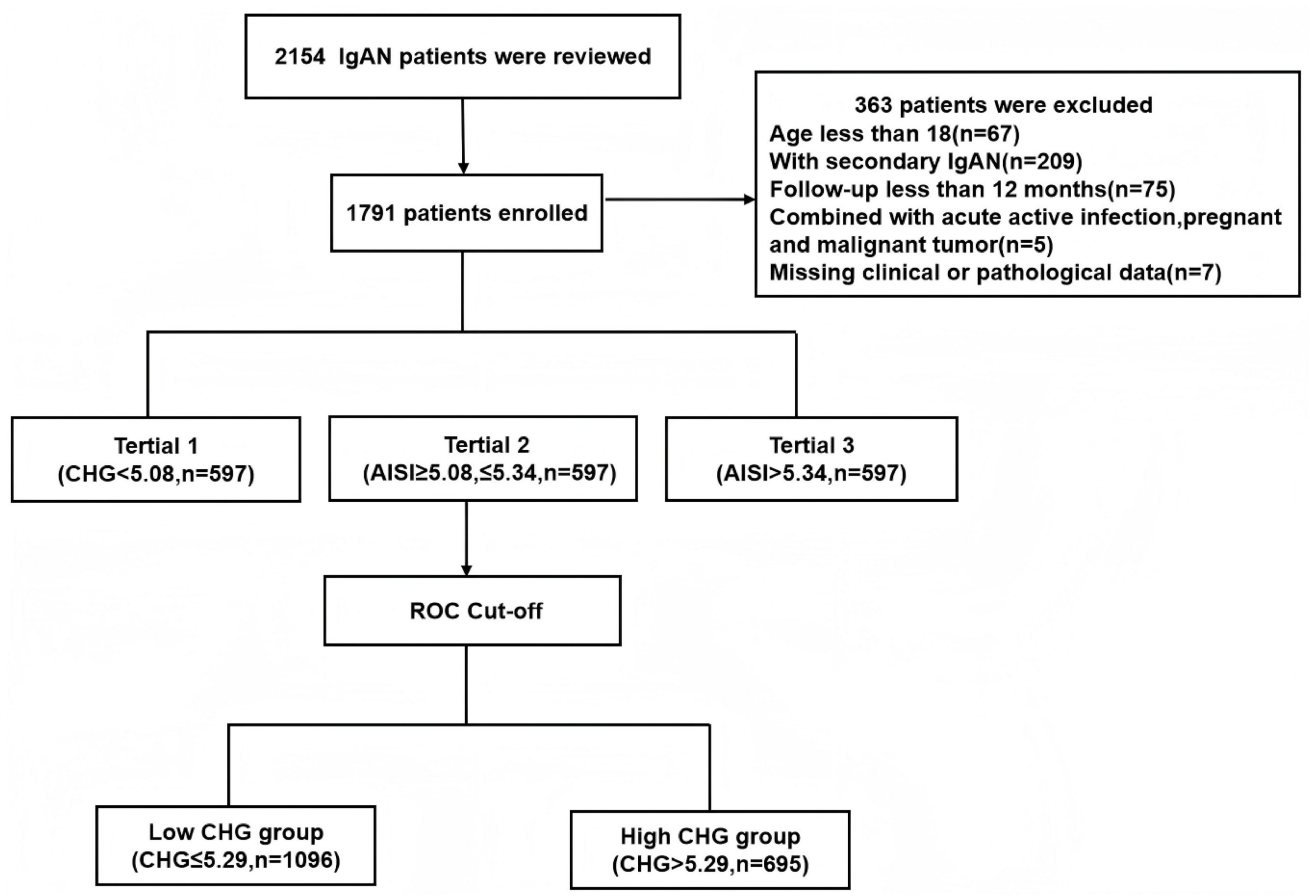
Flowchart of excluded patients.

This single-centre retrospective cohort study included 2,154 patients with biopsy-confirmed IgAN from Hangzhou Hospital of Traditional Chinese Medicine between October 2014 and September 2023. The exclusion criteria were as follows: 1) age under 18 years (n=67); 2) secondary IgAN, including cases associated with diabetes mellitus, Henoch–Schönlein purpura, or autoimmune diseases (n=209); 3) a follow-up duration of less than 12 months (n=75); 4) acute comorbidities such as active infection, pregnancy, or malignancy (n=5); and 5) missing essential clinical or pathological data (n=7). After exclusions, a total of 1,791 eligible patients were included, all of whom had completed at least 12 months of follow-up (**Figure 1**). The study protocol was approved by the Research Ethics Committee of Hangzhou Hospital of Traditional Chinese Medicine (Approval No. 2024KLL230). The committee waived the requirement for written informed consent, as the study utilized only deidentified clinical data and involved no direct patient intervention.

### Clinical data

At the time of kidney biopsy, baseline clinical data were gathered systematically from the hospital’s electronic medical records. The collected data included demographic details (age, sex and BMI), existing health conditions (hypertension and smoking history), and various laboratory measurements (including haemoglobin (Hb), albumin (ALB), total cholesterol (TC), triglyceride (TG), low-density lipoprotein (LDL), high-density lipoprotein (HDL), fasting blood glucose (FBG), uric acid (UA), serum creatinine (Scr), and 24-hour urine protein (24h Upro) levels and the estimated glomerular filtration rate (eGFR)). Additionally, the treatment strategy at the time of the renal biopsy was documented. Renal biopsy specimens were analysed by two skilled pathologists and nephrologists using light microscopy, immunofluorescence, and electron microscopy. The evaluation followed the Oxford classification (MEST-C) for IgAN, which includes assessments of mesangial cellularity (M0/M1), endocapillary proliferation (E0/E1), segmental sclerosis (S0/S1), tubulointerstitial damage (T0/T1/T2), and crescent formation (C0/C1/C2). The inter-rater agreement between the two pathologists for each MEST-C score was excellent, with kappa values of M (κ=0.77), E (κ=0.72), S (κ=0.75), T (κ=0.74), and C (κ=0.76).

### Treatment and definitions

All patients received optimal supportive treatment, which was primarily guided by the KDIGO guidelines and tailored according to the clinical and pathological characteristics of the patients. The treatment regimens included angiotensin-converting enzyme inhibitors (ACEIs) or angiotensin receptor blockers (ARBs), steroids and immunosuppressants such as cyclophosphamide, mycophenolate, tacrolimus, or cyclosporine. The CHG index was calculated by using the following formula: Ln[TC (mg/dL)×FBG (mg/dL)/2×HDL (mg/dL). The eGFR was calculated using the Chronic Kidney Disease Epidemiology Collaboration (CKD-EPI) equation.

### Prognosis definition

The main renal endpoint was established as a combined event, consisting of either a 50% reduction in the eGFR or the onset of ESRD, which was characterized by an eGFR<15 mL/min/1.73 m^2^ or the requirement for kidney replacement therapy.

### Statistical analysis

IBM SPSS software (version 27.0) was used for all the statistical analyses. Non-normally distributed continuous variables are expressed as interquartile ranges and were compared with the Kruskal–Wallis test (for tertiles) or Mann–Whitney U test (for two groups). Categorical data were summarized as frequencies and were analysed using either the χ² test or Fisher’s exact test, as appropriate. The median follow-up time was calculated using the reverse Kaplan–Meier method. Correlation analyses were performed to evaluate the relationships between the CHG index and clinicopathological variables; Spearman correlation analysis was applied for continuous variables, while logistic regression analysis was used for categorical variables. Survival analysis for the renal endpoint was conducted using the Kaplan–Meier method, with differences assessed by the log-rank test. Cox proportional hazards regression models were employed to identify independent predictors of IgAN prognosis, and variable selection for the multivariable Cox model was conducted by considering both clinical relevance and statistical significance. Univariate analyses were carried out for all baseline clinical and pathological variables. Variables for which the p value was < 0.05 in the univariate analysis or recognized clinical significance in IgAN prognosis (such as the eGFR, proteinuria, and MEST-C) were incorporated into the initial comprehensive model. Subgroup analyses were also performed using Cox regression. The cut-off values and predictive performance of CHG, TG/HDL and TC/HDL for renal outcomes were evaluated via receiver operating characteristic (ROC) curve analysis. Additionally, multivariable ROC curve analysis based on logistic regression was conducted to investigate risk factors associated with IgAN renal prognosis. A P value < 0.05 was considered to indicate statistical significance.

## Results

### Baseline characteristics of all participants

A total of 2,154 patients with IgAN were initially identified, but 363 individuals were excluded based on the following criteria: age under 18 years, secondary IgAN, follow-up duration of less than 12 months before reaching endpoints, presence of active infection, pregnancy, malignant tumours, or insufficient pathological data. After strict inclusion and exclusion criteria were applied, 1791 patients were ultimately included in this study, as illustrated in **Figure 1**. The median follow-up for the entire cohort was 45.5 months (range: 12.0–110.0 months). During this period, 130 primary endpoint events were recorded, the majority of which (96 events, 73.8%) occurred after the first 12 months. This cohort consisted of 760 males (42.4%) and 1031 females (57.6%), with a mean age of 43 (interquartile range: 36–53) years. Based on baseline CHG levels, all patients were stratified into three tertiles: tertile 1 (< 5.08, n=597, 33.3%), tertile 2 (≥ 5.08 to ≤ 5.34, n=597, 33.3%), and tertile 3 (> 5.34, n=597, 33.3%). Analysis of baseline characteristics revealed significant differences among the groups for most clinical indicators, except for the presence of hypertension and the use of lipid-lowering drugs. As CHG indices increased, total cholesterol (TCH), triglyceride (TG), low-density lipoprotein (LDL), fasting blood glucose (FBG), serum creatinine (Scr), uric acid (UA), high-sensitivity C-reactive protein (H-CRP), and 24-hour urine protein (24h Upro) levels gradually increased, whereas albumin (ALB) levels, high-density lipoprotein (HDL) levels, and estimated glomerular filtration rates (eGFRs) progressively decreased. Renal pathology MEST-C scores significantly differed across all groups in terms of T and C scores. Additionally, as CHG indices increased, the proportion of patients receiving steroid therapy with or without immunosuppressants also gradually increased, as detailed in **Table 1**.

**Table 1:** Baseline characteristics of 1791 IgAN patients.

Variables	Total (n=1791)	Tertile1 (n=597) <5.08	Tertile2 (n=597) ≥5.08,≤5.34	Tertile3 (n=597) >5.34	P value
Age, years	43(36-53)	40(34-48)	44(36-53)	46(38-57)	**<0.001**
Male, n (%)	760(42.4)	163(27.3)	255(42.7)	342(57.3)	**<0.001**
BMI, (kg/m^2^)	23.05(20.85 -25.35)	21.19(19.4823.27)	23.12(21.1124.13)	24.77(22.6726.84)	**<0.001**
Hypertension, n (%)	195(10.9)	64(10.7)	57(9.5)	74(12.4)	0.284
Smoking, n (%)	172(9.6)	27(4.5)	63(10.6)	82(13.7)	**<0.001**
Hemoglobin, g/L	126.0(113.0 -139.0)	122.0(111.0134.0)	125.5(112.5138)	132.0(117.0144.0)	**<0.001**
Albumin, g/L	38.50(35.80 -41.00)	39.00(36.5041.40)	38.40(35.7040.80)	38.30(34.5040.90)	**0.002**
TC, mmol/L	4.65(4.055.37)	4.23(3.72-4.87)	4.63(4.11-5.30)	5.10(4.535.96)	**<0.001**
TG, mmol/L	1.29(0.901.97)	0.88(0.69-1.19)	1.29(0.95-1.82)	2.02(1.482.72)	**<0.001**
LDL, mmol/L	2.84(2.343.42)	2.39(1.96-2.83)	2.87(2.48-3.38)	3.29(2.814.00)	**<0.001**
HDL, mmol/L	1.08(0.921.30)	1.26(1.07-1.47)	1.07(0.92-1.25)	0.97(0.851.14)	**<0.001**
FBG, mmol/L	4.67(4.305.11)	4.35(4.05-4.68)	4.63(4.31-4.99)	5.13(4.765.66)	**<0.001**
H-CRP, mg/L	1.03(0.512.38)	0.68(0.38-1.60)	0.99(0.51-2.11)	1.50(0.77-3.18)	**<0.001**
UA, umol/L	365(300-437)	321(263-385)	369(305-437)	408(347-478)	**<0.001**
Scr, umol/L	80(62-109)	76(60-99)	81(62-110)	84(64-127)	**<0.001**
24h Upro, g/L	0.91(0.451.80)	0.62(0.33-1.13)	0.93(0.47-1.80)	1.35(0.66-2.94)	**<0.001**
eGFR, ml/min/1.73m2	85.20(61.00106.60)	97.45(73.80115.05)	84.00(61.75104.15)	72.60(49.7096.90)	**<0.001**
Mesangial cellularity, n (%)					
M0	22(1.2)	5(0.8)	6(1.0)	11(1.8)	0.240
M1	1769(98.8)	592(99.2)	591(99.0)	586(98.2)	
Endocapillary hypercellularity, n (%)					
E0	1232(68.8)	419(70.2)	401(67.2)	412(69.0)	0.526
E1	559(31.2)	178(29.8)	196(32.8)	185(31.0)	
Segmental sclerosis, n (%)					
S0	345(19.3)	112(18.8)	105(17.6)	128(21.4)	0.224
S1	1446(80.7)	485(81.2)	492(82.4)	469(78.6)	
Tubular atrophy/interstitial fibrosis, n (%)					
T0	1255(70.1)	483(80.9)	409(68.5)	363(60.8)	**<0.001**
T1	414(23.1)	105(17.6)	140(23.5)	169(28.3)	
T2	122(6.8)	9(1.5)	48(8.0)	65(10.9)	
Crescents, n (%)					
C0	642(35.8)	232(39.0)	185(31.0)	224(37.5)	**0.044**
C1	1004(56.1)	321(53.8)	359(60.1)	324(54.3)	
C2	145(8.1)	43(7.2)	53(8.9)	49(8.2)	
Using SGLT2i drugs, n (%)	383(21.4)	125(20.9)	119(20.0)	139(23.3)	0.350
Using ACEI/ARB drugs, n (%)	1401(78.2)	453(75.9)	471(78.9)	477(79.9)	0.216
Using lipid-lowering drugs	151(8.4)	45(7.5)	54(9.0)	52(8.7)	0.612
Steroid with/without immunosuppressants treatment, n (%)	512(28.6)	138(23.1)	184(30.8)	190(31.8)	**0.001**
Composite event, n (%)	130(7.3)	23(3.9)	41(6.9)	66(11.1)	**<0.001**

Continuous variables are expressed as median (interquartile range); Categorical variables are expressed as frequency (%); Bold values was that the differences were significant (P<0.05).

BMI, body mass index; TC, total cholesterol; TG, triglyceride; LDL, low-density lipoprotein; HDL, high-density lipoprotein; FBG, fasting blood glucose; UA, uric acid ;Scr, serum creatinine; 24h Upro, 24-hour urine protein; eGFR, estimated glomerular filtration rate; SGLT2i, sodium-glucose cotransporter-2 inhibitors; ACEI/ARB, angiotensin-converting enzyme inhibitors/angiotensin II receptor blockers.

### Correlation of the CHG index with clinical parameters and pathological lesions

Correlation analyses were carried out to investigate the associations between the CHG index and critical clinicopathological factors. As indicated in **Table 2**, our results revealed strong positive relationships between the CHG index and Hb (r = 0.202, P<0.001), Scr (r = 0.297, P<0.001), UA (r = 0.370, P<0.001), and 24h Upro (r = 0.341, P<0.001) levels. In contrast, CHG exhibited a significant negative association with ALB level (r = -0.090,P<0.001) and the eGFR (r = -0.306,P<0.001). Furthermore, logistic regression analysis was performed to explore the associations between the CHG index and pathological features. The findings displayed in **Table 3** confirmed the statistical significance of T1–2 compared with T0, with an odds ratio (OR) of 3.489 (95% CI: 2.494–4.882, P< 0.001), and the CHG index was positively correlated with both intimal thickening and hyaline degeneration of renal arteries (P<0.001); specifically, for each unit increase in the CHG level, compared with controls, patients with IgAN had a 2.486-fold greater risk of arterial intimal thickening and a 4.513-fold greater risk of vascular hyalinosis. Moreover, individuals with a high CHG index showed a higher likelihood of being smokers (OR = 3.527, 95% CI: 2.179–5.708, P<0.001) and experiencing reduced renal function (eGFR < 45mL/min/1.73m²; OR = 4.593; 95% CI: 3.050–6.916; P<0.001).

**Table 2:** Correlation between CHG index and potential risk factors.

	Variables	Correlation coefficient(r)	P value
CHG	sex	0.258	**<0.001**
	age	0.223	**<0.001**
	BMI	0.431	**<0.001**
	Hb	0.202	**<0.001**
	ALB	-0.090	**<0.001**
	UA	0.370	**<0.001**
	Scr	0.297	**<0.001**
	24h Upro	0.341	**<0.001**
	eGFR	-0.306	**<0.001**

Bold values was that the differences were significant (P<0.05).

CHG, the Cholesterol, High-Density Lipoprotein, and Glucose (CHG) index; BMI, body mass index; Hb, hemoglobin; ALB, albumin; UA, uric acid; Scr, serum creatinine; 24h Upro, 24-hour urine protein; eGFR, estimated glomerular filtration rate.

**Table 3:** Logistic Regression Models for the relationship between CHG index and renal pathological lesions and clinical manifestation.

Pathological lesions	OR	95%CI	P value
M1/M0	0.338	0.099-1.151	0.083
E1/E0	1.183	0.860-1.629	0.302
S1/S0	0.861	0.592-1.253	0.435
T1-2/T0	3.489	2.494-4.882	**<0.001**
C1-2/C0	1.062	0.779-1.448	0.705
Intimal thickening of artery	2.486	1.795-3.443	**<0.001**
Hyaline degeneration	4.513	3.231-6.305	**<0.001**
Smoking	3.527	2.179-5.708	**<0.001**
Hypertension	1.176	0.733-1.887	0.502
eGFR<45 ml/min/1.73m^2^	4.593	3.050-6.916	**<0.001**

Bold values was that the differences were significant (P<0.05).

M, mesangial proliferation; E, endocapillary proliferation; S, segmental glomerulosclerosis; T, tubular atrophy or interstitial fibrosis; C, crescents.

### Renal survival

**Figure 2:**
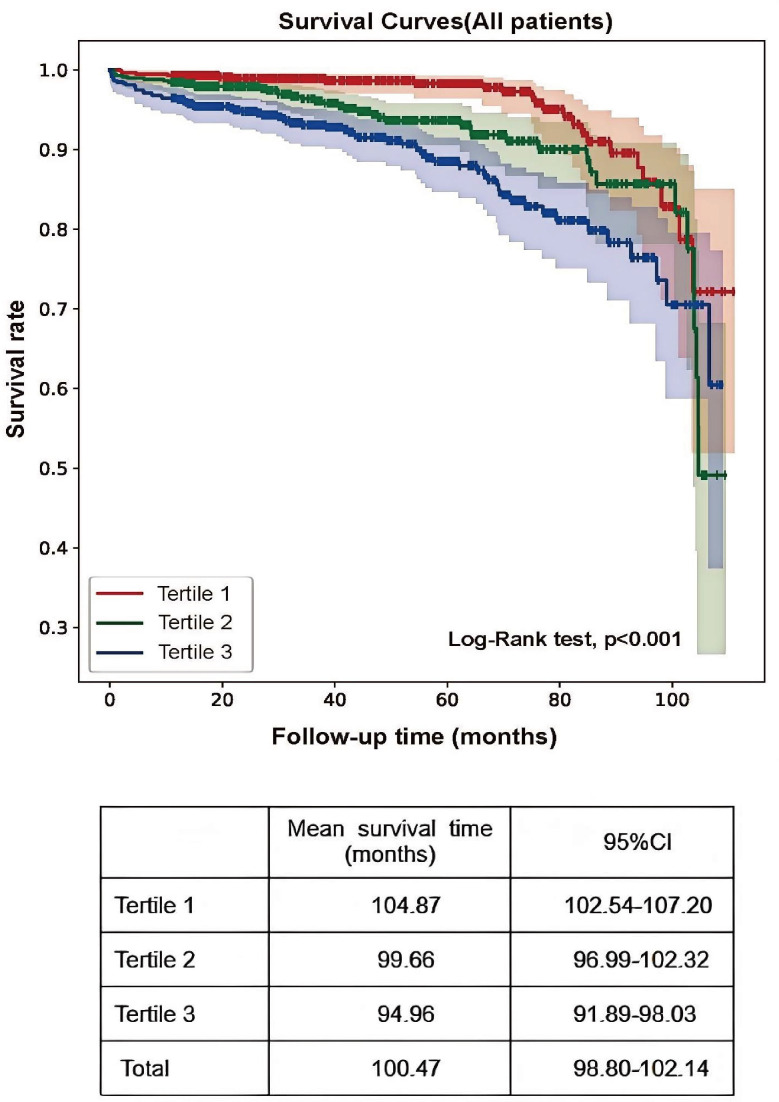
Kaplan–Meier curves of renal outcomes in different CHG groups.

The primary renal endpoint was defined as a 50% decrease in the eGFR or the development of ESRD. Analysis revealed that 11.1% of participants in tertile 3 reached the endpoint, compared with 6.9% and 3.9% in the lower tertiles (P<0.001; **Table 1**). Kaplan–Meier survival analysis revealed a significantly greater risk of kidney failure in tertile 3 (log-rank = 26.51; P<0.001), with patients in tertile 1 exhibiting markedly longer mean renal survival (104.87 months; 95% CI: 102.54–107.20) than the other groups (**Figure 2**).

### Predictive value of the CHG index for IgA nephropathy renal outcomes

**Figure 3:**
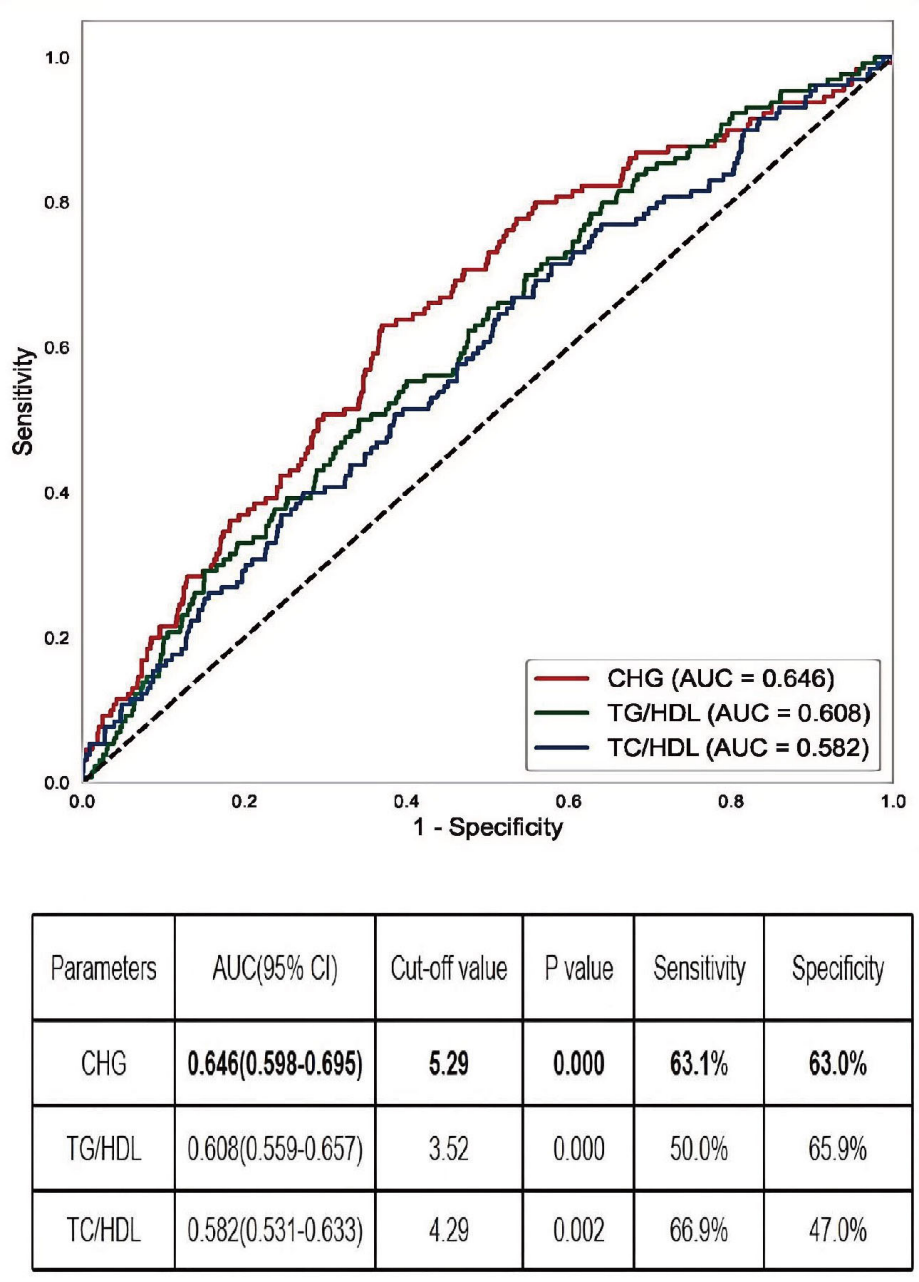
The AUC of CHG, TG/HDL, and TC/HDL for IgAN renal outcomes. AUC, the area under the receiver operating characteristic curve; CHG, the Cholesterol, High-Density Lipoprotein, and Glucose = Ln[TC (mg/dL) × FBG (mg/dL)/2×HDL (mg/dL).

ROC curve analysis was performed to evaluate the ability of CHG to predict renal outcomes in IgAN patients. The CHG index demonstrated a slightly superior predictive value (AUC = 0.646) compared with both TG/HDL levels (AUC = 0.608) and TC/HDL levels (AUC = 0.582); however, its overall predictive accuracy remained moderate. The optimal CHG index cut-off value was 5.29, with a sensitivity of 63.1% and a specificity of 63.0% (**Figure 3**).

### CHG index as an independent risk factor for the progression of IgAN to ESRD

**Figure 4:**
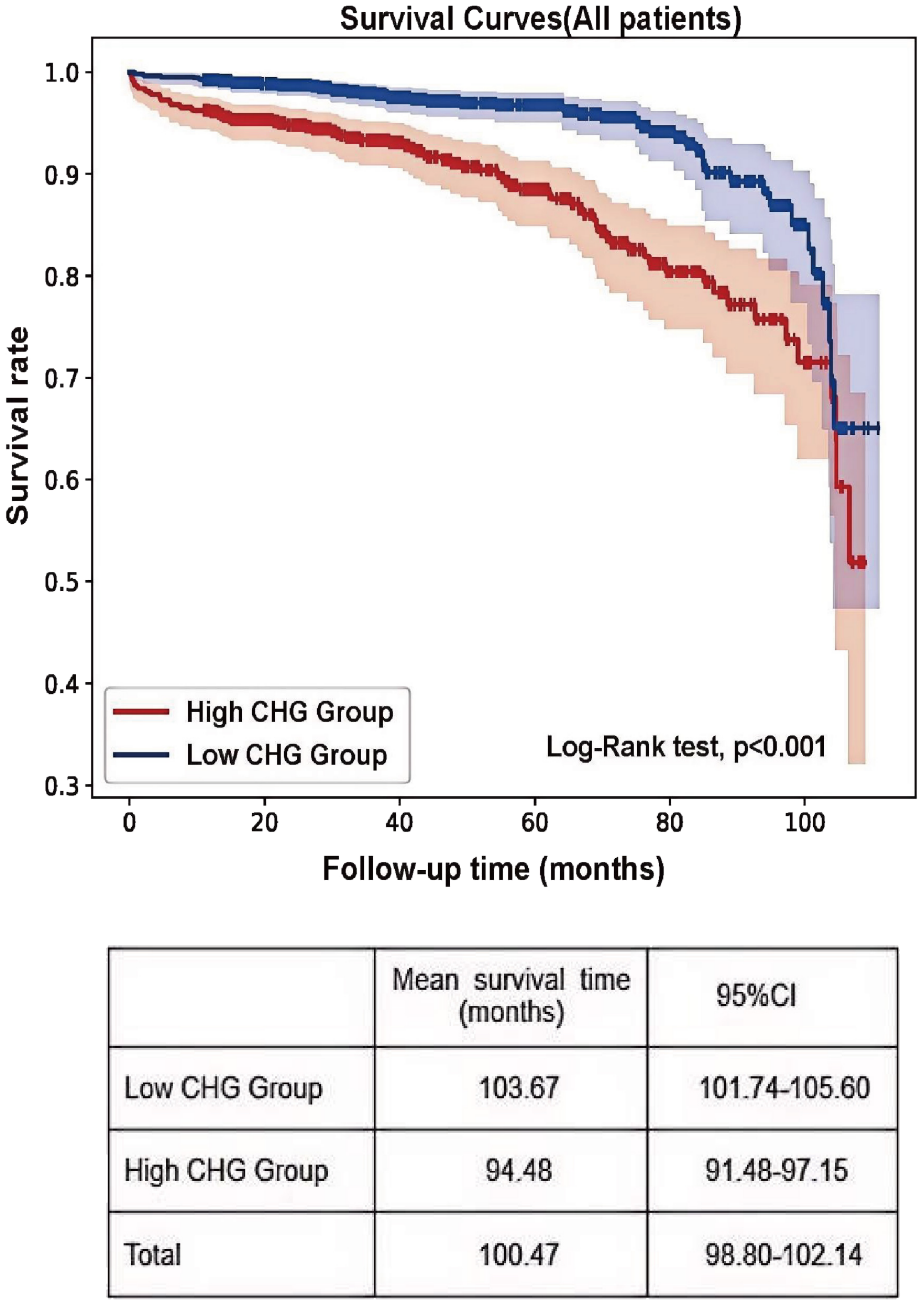
Kaplan–Meier curves of renal outcomes in two CHG groups.

Using the established CHG index cut-off value, the cohort was stratified into high (CHG index > 5.29) and low (CHG index ≤ 5.29) groups. As shown in **Table 4**, numerous baseline variables exhibited statistically significant differences between the two groups, and the proportion of patients in the high CHG group who reached the renal endpoint was significantly higher (P < 0.001). K–M analysis revealed a significantly greater risk of kidney failure in the high-CHG index group (log-rank=31.569, P<0.001), with the low-CHG index group exhibiting a notably longer mean renal survival (**Figure 4**). In the Cox regression analysis, CHG indices were categorized based on a predefined cut-off value. Unadjusted Cox modelling revealed a significantly greater risk of adverse renal outcomes in the high-CHG index group (HR = 2.666;95% CI:1.868–3.804;P<0.001). To account for potential confounding factors, three separate statistical models were constructed, incorporating a comprehensive set of clinical, pathological, and treatment-related variables. Multivariate Cox regression analysis, as detailed in Models 1, 2, and 3, indicated that elevated CHG indices remained a significant independent predictor of renal disease progression. After adjustment for clinical parameters, including age; sex; BMI; Hb, ALB,UA, and 24h Upro levels; and eGFR, the adjusted HR was 1.986 (95% CI:1.142–3.454; P = 0.0015). Consistent findings were observed when pathological characteristics were integrated according to the Oxford MEST-C (adjusted HR:1.853; 95% CI:1.047–3.280; P = 0.034) and when treatment variables were accounted for (adjusted HR:1.842; 95% CI:1.044–3.249;P=0.035), as summarized in **Table 5**.

**Table 4:** Baseline characteristics of 1791 IgAN patients according to the Cut-off value of CHG.

Variables	Total	Low CHG group (≤5.29)	High CHG group (>5.29)	P value
Participants, (n)	1791	1096	695	
Age,years	43(36-53)	41(35-50)	47(38-57)	**<0.001**
Male, n (%)	760(42.4)	367(33.5)	393(56.5)	**<0.001**
BMI, (kg/m^2^)	23.05(20.8525.35)	22.05(20.0724.14)	24.61(22.4926.70)	**<0.001**
Hypertension, n (%)	195(10.5)	112(10.2)	83(11.9)	0.254
Smoking, n (%)	172(9.6)	76(6.9)	96(13.8)	**<0.001**
Hemoglobin, g/L	126(113-139)	123(112-135)	131(117-144)	**<0.001**
Albumin, g/L	38.5(35.8-41.0)	38.7(36.2-41.2)	38.4(34.6-40.9)	**0.011**
TC, mmol/L	4.65(4.05-5.37)	4.39(3.86-5.05)	5.07(4.49-5.93)	**<0.001**
TG, mmol/L	1.29(0.90-1.97)	1.02(0.77-1.41)	1.96(1.43-2.64)	**<0.001**
LDL, mmol/L	2.84(2.34-3.42)	2.58(2.16-3.03)	3.25(2.79-3.96)	**<0.001**
HDL, mmol/L	1.08(0.92-1.30)	1.16(0.98-1.38)	0.98(0.85-1.16)	**<0.001**
FBG, mmol/L	4.67(4.30-5.11)	4.45(4.14-4.81)	5.07(4.74-5.58)	**<0.001**
H-CRP, mg/L	1.03(0.51-2.38)	0.83(0.42-1.85)	1.43(0.73-3.02)	**<0.001**
UA, umol/L	365(300-437)	336(280-408)	406(345-476)	**<0.001**
Scr, umol/L	80(62-109)	73.0(59.0-96.7)	95(71-131)	**<0.001**
24h Upro, g/L	0.91(0.45-1.80)	0.69(0.37-1.30)	1.39(0.69-2.82)	**<0.001**
eGFR, ml/min/1.73m^2^	85.2(61.0-106.6)	92.7(70.7-111.0)	71.5(49.6-94.1)	**<0.001**
Mesangial cellularity, n (%)				
M0	22(1.2)	9(0.8)	13(1.9)	**0.049**
M1	1769(98.8)	1087(99.2)	682(98.1)	
Endocapillary hypercellularity, n (%)				
E0	1232(68.8)	754(68.8)	478(68.8)	0.993
E1	559(31.2)	342(31.2)	217(31.2)	
Segmental sclerosis, n (%)				
S0	345(19.3)	200(18.2)	145(20.9)	0.171
S1	1446(80.7)	896(81.8)	550(79.1)	
Tubular atrophy/interstitial fibrosis, n (%)				
T0	1225(70.1)	844(77.0)	411(59.1)	**<0.001**
T1-2	536(29.9)	252(23.0)	284(40.9)	
Crescents, n (%)				
C0	642(35.8)	386(35.2)	256(36.8)	0.487
C1-2	1149(64.2)	710(64.8)	439(63.2)	
Steroid with/without immunosuppressants treatment, n (%)	512(28.6)	288(26.3)	224(32.2)	**0.007**
Composite event, n (%)	130(7.3)	49(4.5)	81(11.7)	**<0.001**

Continuous variables are expressed as median (interquartile range); Categorical variables are expressed as frequency (%); Bold values was that the differences were significant (P<0.05).

BMI, body mass index; TC, total cholesterol; TG, triglyceride; LDL, low-density lipoprotein; HDL, high-density lipoprotein; FBG, fasting blood glucose; UA, uric acid; Scr, serum creatinine; 24h Upro, 24-hour urine protein; eGFR, estimated glomerular filtration rate.

**Table 5:** Multivariate Cox regression analysis CHG according to Cut-off value and renal outcomes.

	Low CHG group (≤5.29)	High CHG group (>5.29)	P value
Number of participants with events/n	49/1096	81/695	
Crude Model	1.00(reference)	2.666 (1.868-3.804)	**<0.001**
Model 1	1.00(reference)	1.986 (1.142-3.454)	**0.015**
Model 2	1.00(reference)	1.853 (1.047-3.280)	**0.034**
Model 3	1.00(reference)	1.842 (1.044-3.249)	**0.035**

Model 1: was adjusted for age, gender, BMI+ clinic factors (hemoglobin, albumin, uric acid, 24h Upro and eGFR ). Model 2: was adjusted for Model 1 +Oxford(MEST-C). Model 3: was adjusted for Model 2 + treatment. albumin was transformed into a binary variable with a cutoff of 35.uric acid was transformed into a binary variable with a cutoff of 420.eGFR was transformed into a binary variable with a cutoff of 45. Tubulointerstitial atrophy/interstitial fibrosis (T) was transformed into a binary variable of T0 and T1+T2. Crescent(C) was transformed into a binary variable of C0 and C1+C2. CI, confidence intervals; HR, hazard ratios. Bold values was that the differences were significant.

### Subgroup analysis

**Figure 5:**
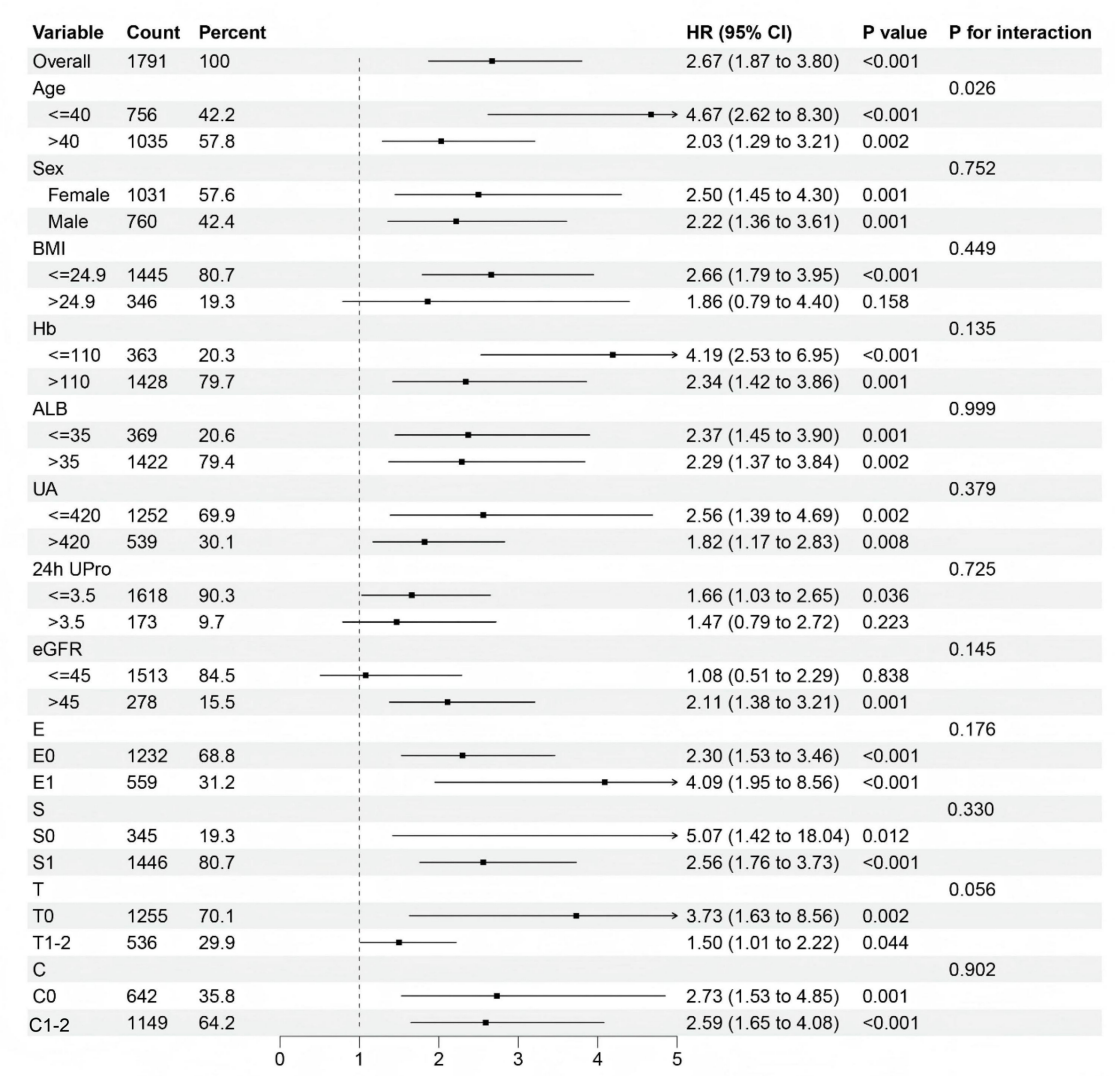
Forest plot of subgroup and interaction effects analyses.

**Figure 6:**
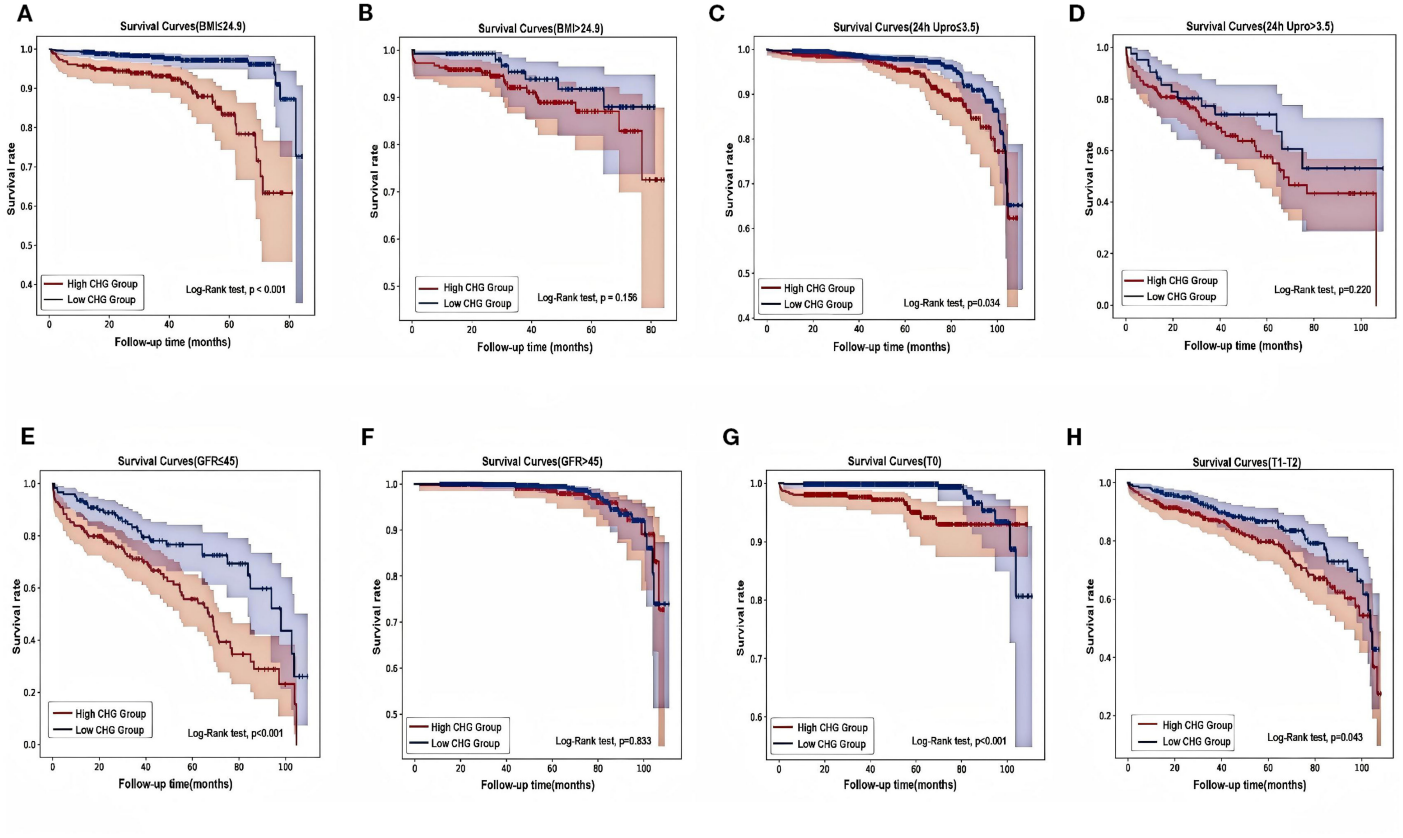
Different types of Kaplan-Meier analysis for the renal endpoint. **(A, B)** Kaplan-Meier analysis for with different BMI. **(C, D)** Kaplan-Meier analysis for patients with different 24-hour urine protein. **(E, F)** Kaplan-Meier analysis for patients with different eGFR. **(G, H)** Kaplan-Meier analysis for patients with differenttubulointerstitial atrophy/interstitial fibrosis (T). BMI, body mass index; 24h Upro, 24h urine protein; eGFR, estimated glomerular filtration rate; T, Tubular atrophy/interstitial fibrosis.

The results of subgroup analyses evaluating the predictive value of the CHG index for renal endpoint events across different patient subgroups are shown in **Figure 5**. Notably, the CHG index demonstrated enhanced predictive accuracy in patients with a BMI ≤ 24.9, 24-hour urine protein level ≤ 3.5g, an eGFR ≤ 45mL/min/1.73m², and Oxford MEST-C T0 pathology. Kaplan–Meier survival analyses (**Figure 6**) further validated these findings, revealing significantly stronger associations in the aforementioned subgroups (BMI ≤ 24.9: log-rank=32.733, P<0.001; 24h Upro level ≤ 3.5g: log-rank=4.503, P = 0.034; eGFR ≤ 45mL/min/1.73m²: log-rank=12.591, P<0.001; T0: log-rank=11.125, P<0.001).

### Multivariate ROC analysis of the CHG-integrated IIgA-PRT model

**Figure 7:**
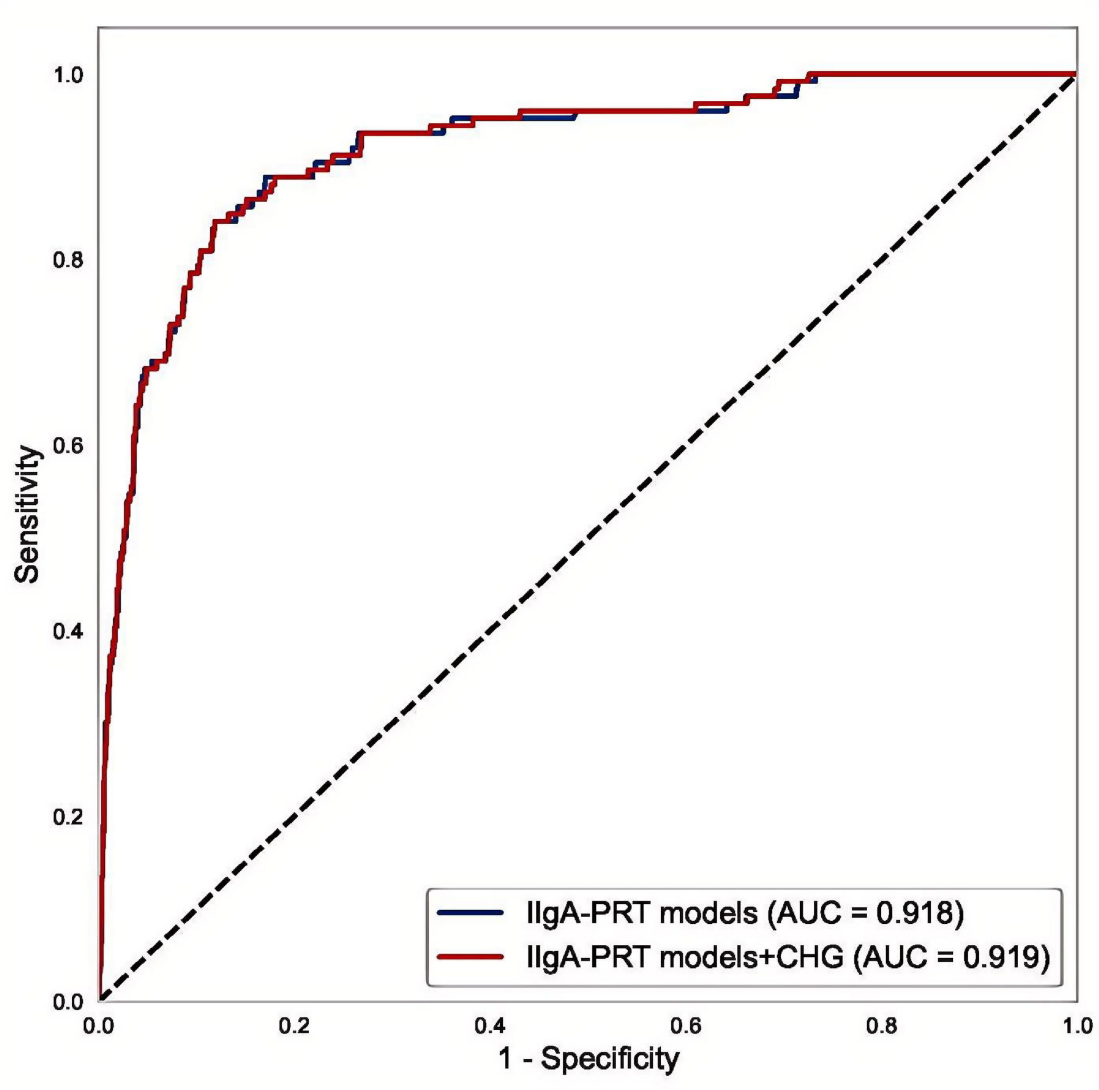
The ROC of IIgAN-PRT models with and without CHG.

Multivariate logistic regression analysis was performed on the initial IIgAN-PRT model, considering relevant parameters in both the presence and absence of the CHG index. The results of the ROC analysis indicated that compared with the original model (AUC = 0.918), the model with the CHG index (AUC = 0.919) had a marginally higher AUC, as illustrated in **Figure 7**.

## Discussion

IgA nephropathy (IgAN) is a chronic immune-mediated inflammatory disease, the pathogenesis of which remains complex and not fully elucidated. Accumulating evidence has established hypertension (10), proteinuria (11), hyperuricaemia (12), abnormal glucose metabolism (13), and dyslipidaemia (14) as risk factors for IgAN progression. The prevalence of metabolic abnormalities in patients with IgAN is strongly correlated with the severity of clinical manifestations and poor renal outcomes (15). The CHG index has emerged as a robust biomarker with significant potential for screening and predicting various clinical conditions related to insulin resistance (IR) and metabolic dysfunction (8, 9); however, its relationship with IgAN progression remains unknown.

This retrospective study, which included 1,791 patients, demonstrated a strong positive association between the CHG index and IgAN progression. Patients with elevated CHG indices exhibited more severe clinical and pathological profiles, along with a higher risk of composite endpoint events. Correlation analyses indicated that the CHG index was negatively correlated with the eGFR and positively correlated with UA, Scr, and 24h Upro levels. Histopathological assessment revealed more severe lesions in the high-CHG index group, including tubular atrophy/interstitial fibrosis and renal arterial intimal thickening with hyaline degeneration. Kaplan–Meier survival analysis revealed that the mean renal survival time was significantly shorter in the high-CHG index group than in the low-CHG index group. To our knowledge, this is the first study to investigate the association between the CHG index, a novel marker of metabolic dysfunction, and renal progression in patients with IgAN.

The precise mechanism through which elevated CHG levels affect the prognosis of IgAN remains unclear; however, the underlying pathways are thought to involve insulin resistance (IR), oxidative stress, and inflammatory responses. First, dyslipidaemia and hyperglycaemia are interrelated mechanisms that contribute significantly to the development of IR (16); as a novel marker for assessing IR, the CHG index includes only metabolic variables (TC, HDL and FBG levels). Emerging evidence links dyslipidaemia to both disease progression and poor prognosis in IgAN, identifying it as a key risk factor for ESRD (13), and hypercholesterolemia and low high-density lipoproteinaemia contribute to renal injury by inducing damage to glomerular endothelial and mesangial cells and promoting inflammatory cell infiltration, renal atherosclerosis, and tubular atrophy/interstitial fibrosis (17). Ma H et al. (7) reported that fasting blood glucose (FBG) levels are positively correlated with increased 24-hour urinary protein excretion and decreased eGFR in IgAN patients and that elevated FBG levels promote renal oxidative stress through pathways involving advanced glycation end products (AGEs) and protein kinase C (PKC), which drive mesangial cell proliferation, matrix secretion, and podocyte injury, ultimately leading to renal injury (18). Moreover, IR can exacerbate preexisting glucose and lipid metabolic abnormalities, thereby promoting advanced glycation and lipotoxicity that trigger inflammatory responses and oxidative stress, ultimately leading to fibroblast proliferation and collagen deposition (19). As demonstrated in our study, the CHG index was closely associated with tubulointerstitial sclerosis, arterial intimal thickening, and hyaline degeneration. Third, both serum lipid levels and glucose levels have been shown to correlate with the degree of inflammation. Peng L et al. (20) demonstrated that reducing TC levels significantly ameliorates inflammatory infiltration and fibrotic changes in renal allografts, and an *in vitro* cell study demonstrated that high glucose levels suppress human renal mesangial cell survival by upregulating proinflammatory cytokine expression (21); subsequently, theoretically, the CHG index could be used to evaluate the inflammatory response. However, no current studies have definitively confirmed this hypothesis.

Recently, TG/HDL and total TC/HDL ratios have been validated as biomarkers for predicting type 2 diabetes, cardiovascular disease, and metabolic syndrome (22, 23), with established prognostic relevance in IgAN (6, 24). Our study demonstrated a slightly superior capacity of the CHG index (AUC = 0.646) compared with TG/HDL(AUC = 0.608) and TC/HDL(AUC = 0.582) ratios for predicting renal outcomes in patients with IgAN. Despite its statistical significance and superiority to traditional lipid ratios, the predictive ability of the CHG index is limited. An AUC of 0.646, although acceptable for an initial investigation, indicates only moderate discriminatory power. As a novel biomarker of insulin resistance and metabolic dysfunction, the CHG index has limited independent prognostic value for IgAN. Nonetheless, it functions as a valuable complementary biomarker that enhances the predictive accuracy of the established IIgAN-PT model, as evidenced by our results.

When the optimal CHG cut-off value of 5.29 was used, stratification revealed that patients with a CHG index > 5.29 had significantly higher proteinuria levels, lower eGFRs, and more advanced pathological lesions, collectively indicating an increased risk of disease progression. Multivariate Cox regression confirmed high CHG index (>5.29) as an independent predictor of adverse renal outcomes (adjusted HR:1.842; 95% CI: 1.044–3.249; P = 0.035). Subgroup analyses further revealed that the prognostic impact of CHG was particularly pronounced in patients with a BMI ≤ 24.9 kg/m², 24-hour urine protein level ≤ 3.5g, and an eGFR ≤ 45mL/min/1.73m². While the exact mechanisms warrant further investigation, we consider that metabolic abnormalities are prevalent in obese patients (BMI>24.9) and in patients with high proteinuria (24-hour urinary protein level > 3.5g) and that nonmetabolic factors such as glomerular hyperperfusion, hyperfiltration, podocyte injury, and immune-inflammatory responses may be major drivers of IgAN progression and that CHG, as a metabolic marker, contributes less of a predictive value in these subgroups. These findings indicate that elevated CHG indices may require enhanced clinical vigilance for early risk stratification in IgAN patients with a normal BMI, non-nephrotic range proteinuria, or moderate renal dysfunction.

The homeostasis model assessment of insulin resistance (HOMA-IR) is the gold-standard index for evaluating IR (25). However, this method is associated with high costs and time consumption, and substantial bias may arise from inaccuracies in insulin measurement (26). The CHG index, which serves as a robust marker for IR and MS, is easy to calculate because its required values can be derived from routine laboratory tests in a minimally invasive, cost-effective, and convenient manner. Furthermore, it may represent a potential intervention target for preventing renal progression.

While our study confirms the association between the CHG index and outcomes in patients with IgAN, several limitations must be acknowledged. First, as a single-centre retrospective study with a relatively short follow-up period conducted in a Chinese population, the findings are susceptible to selection bias, which may limit their generalizability. Future multicentre prospective studies involving diverse geographic and ethnic cohorts are essential to validate our results. Second, the absence of direct measures of IR, such as HOMA-IR, hinders the ability to establish a causal relationship between the CHG index and IR. Third, we did not analyse the impact of dynamic changes in CHG index over time on clinical outcomes—particularly whether reducing CHG levels following intervention could improve prognosis—thereby weakening the evidence supporting its utility as an intervention target. Most importantly, the optimal CHG index cut-off value of 5.29 was derived and internally validated within a single-centre cohort. The lack of external validation in an independent, multicentre population implies that its generalizability and clinical applicability remain uncertain. Therefore, the proposed cut-off value should be considered preliminary and requires prospective validation in larger, external cohorts before any clinical application can be recommended.

## Conclusion

In conclusion, the CHG index, acting as a new and potential indicator for IR and MS, is strongly correlated with severe clinical and pathological features in patients with IgAN. Consequently, it may serve as an independent risk factor for predicting IgAN progression through a minimally invasive and economically feasible approach. For patients with IgAN whose CHG index is greater than 5.29 during renal biopsy, increased monitoring and intensified treatment strategies are recommended.

## Data Availability

The original contributions presented in the study are included in the article/Supplementary Material. Further inquiries can be directed to the corresponding author.
